# Physical and Mental Recovery for Female Footballers: Considerations and Approaches for Better Practice

**DOI:** 10.1007/s40279-025-02246-x

**Published:** 2025-08-25

**Authors:** Glyn Howatson, Suzanna Russell, Charles Pedlar, Shona Halson

**Affiliations:** 1 Department of Sport, Exercise, and Rehabilitation, Faculty of Health and Life Sciences, Northumbria University, Sutherland Building, Newcastle-Upon-Tyne, NE1 8ST UK; 2 Water Research Group, School of Biological Sciences, North-West University, Potchefstroom, South Africa; 3 School of Behavioural and Health Sciences, Australian Catholic University, Brisbane, QLD Australia; 4 Sports Performance, Recovery, Injury and New Technologies (SPRINT) Research Centre, Australian Catholic University, Brisbane, QLD Australia; 5 Faculty of Sport, Technology and Health Sciences, St Mary’s University, Twickenham, London, UK; 6 Institute of Sport, Exercise and Health, Division of Surgery and Interventional Science, University College London, London, UK

## Abstract

Increased physiological demands in elite women’s football coupled with growing demands on and off the field of play have inevitably placed more pressure on players. Recovery therefore plays a critical role in sustaining health and maintaining high performance for training and readiness to compete. Recovery strategies start with the fundamental need for adequate sleep quality and duration, and nutrition. When these are in place, recovery could be further augmented with additional recovery techniques. Where there is a priority to maximise an adaptative response, there is an argument to withhold additional recovery strategies to maximise the adaptation stimulus. Conversely, when rapid recovery is desired for an imminent match, or in a tournament setting, the application of recovery strategies must be prioritised. This article discusses the approaches that should be considered to support physical and mental recovery and regeneration strategies in the context of women’s football. Whilst most recovery research is based on studies of male athletes, there is also work that exclusively focusses on female individuals; this article highlights the potential applicability of this collective work and specific considerations for female football players and offers practical recommendations. Although far from complete, there is emerging evidence of an interplay between cyclical variations of reproductive hormones, associated menstrual cycle/hormonal contraception symptoms and recovery/adaptation. Whilst there is an expected individual variability in menstrual cycle and symptoms, these additional female-specific considerations might contribute to the total stress and recovery needs of the individual athlete. Exploring the role of recovery strategies in support of training and competition for female football players represents an exciting area for future research.

## Key Points

**Table:** 

There is a paucity of information relating to female athletes despite representation in many sports at the elite level, including football, gaining parity. This lack of an evidence base extends to the area of exercise recovery, where female athletes are not well considered.
This article forms a basis for athletes, coaches, practitioners and scientists to approach recovery strategies that can be applied with female football athletes. The fundamentals of physical and mental recovery reside firmly in doing the simple things better with due attention to the potential use of additional interventions. Critical to the individuality of the female athlete, due consideration should be made with regard to the athlete’s psycho-biology.
Despite the lack of female-specific information, practical advice and approaches are presented here. The field of performance recovery in support of female footballers represents an exciting opportunity for research to provide evidence-based practice, but conversely for practitioners to fuel research and their own work through practice-based evidence.

## Background

**Fig. 1:**
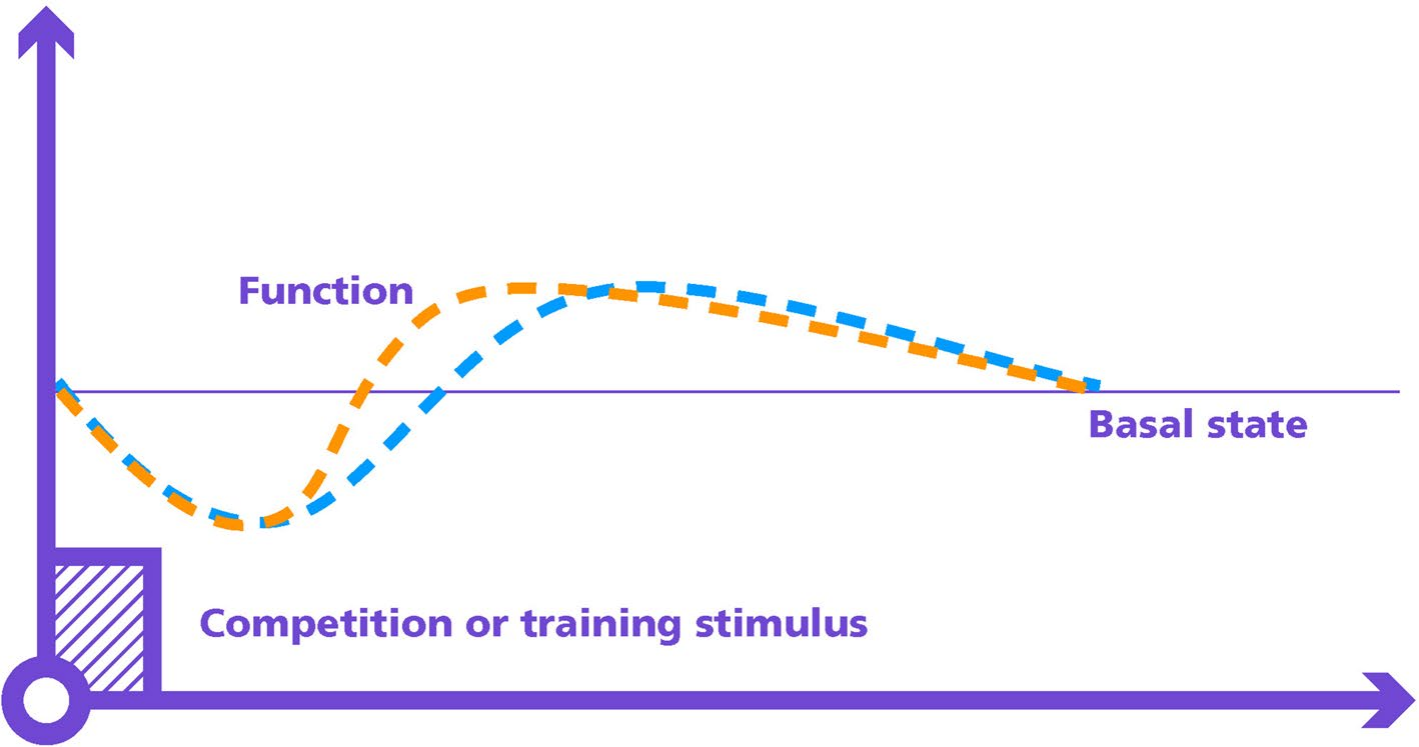
Timeline of the stress-recovery-adaptation continuum (adapted from Selye’s general adaptation syndrome). The *black line* indicates the timeline of unassisted recovery following training or competition, where function is restored, and an adaptive response is observed. If a further stimulus is not introduced to the biological system, the function will return to the basal state. The *dashed grey line* illustrates the concept of a successful recovery strategy where function is accelerated with no loss in the adaptive response

This article provides an overview of the factors that should be considered when facilitating recovery in female footballers. It is not the intention to provide a review of contemporary recovery strategies, but rather the contributing factors influencing the successful application of any recovery intervention. Recovery has become an increasingly important tenet of athletic performance and is central to restoration of a homeostatic state, and critically, to the realisation of physiological adaptation. Notably, in elite women’s football, the physiological requirements have increased, which is evident by escalating competition demands [1], increasing fixture congestion and overall seasonal volume [2]. Consequently, the interest in exercise recovery has never been so important to consider for female football. However, the right balance between the physiological and non-physiological of (non) training and competition stressors, recovery and subsequent adaptation is difficult to optimise. A successful recovery regime could lead to: (1) reduced fatigue and stress induced by training or competition; (2) accelerated recovery times to allow for an additional training stimulus; (3) greater physiological adaptation; and (4) optimised recovery in periods of competition congestion. Conceptually, the idea of the “right” recovery would result in an accelerated return to homeostasis following a training or competition stressor. In an ideal scenario, this would be followed by a period of supercompensation where a positive adaptive response would occur, similar to that described in the General Adaptation Syndrome model [3] and evolved to apply to exercise physiology [4]. In the absence of further stressors, a return to a homeostatic state would be expected (Fig. 1).

In female individuals, the return of homeostasis may be subtlety different to male individuals given the fluctuation of endogenous (and often exogenous through oral contraceptives) reproductive hormones and sex differences [5], which indicates the importance of understanding the physiological implications on an individual basis. These hormone fluctuations (predominantly oestrogen and progesterone) conceptually affect performance and might help to facilitate recovery or conversely prolong or compromise recovery. For an in-depth review of the menstrual cycle and oral contraceptives on performance, the reader is directed to these current reviews [6, 7]. There is evidence (albeit sparse) showing differences during the follicular phase of the menstrual cycle versus the luteal phase, suggesting that recovery might be more effective earlier in the menstrual cycle [8–10]. For example, previous work [11] showed that oestrogen might support membrane integrity and hence reduce the extent of muscle damage (evidenced by lower creatine kinase and interleukin-6; indirect indices of damage and inflammation, respectively) following strenuous exercise. This has the potential to have greater recovery during the mid-luteal phase compared with the mid-follicular phase where progesterone and oestrogen are lower. This is far from unequivocal, and it seems there is wide individual variability and hence generalisation is not recommended. The role of female sex hormones in muscle homeostasis has attracted attention in the sports medicine literature; however, to date, studies in humans have shown inconsistent results [12]. In post-menopause, female individuals have diminished sex hormones and muscle mass, but hormone replacement therapy can be preventative, which offers valuable insights for the important role female sex hormones might play in recovery and adaptation [13].

The phase of the menstrual cycle could be relevant to recovery priorities. For example, the pre-menstrual window (often referred to as ‘phase 4’) is characterised as an inflammatory process [14–16], leading to the breakdown of tissue and a menstrual bleed, and often accompanied by menstrual cycle symptoms that might include, to varying degrees of severity, cramps, brain fog, anxiety, disturbed sleep and pre-menstrual syndrome. Although the degree to which menstrual cycle symptoms might affect female individuals could vary enormously [17], factoring in the menstrual cycle phase into recovery strategy selection and periodisation (see below) has not been systematically studied in athletes, but teleologically could lead to positive performance and health outcomes.

## Doing the Basics Better

Most recovery interventions are likely to yield only modest improvements in recovery but have the potential for a meaningful impact on the athlete. For example, a systematic review with meta-analysis showed compression garments, compared to a control, to have a small effect (Hedges’ *g* = 0.4) on muscle soreness in male and female individuals, but the effect is likely to be seen in 66% of those who use it [18]. It is critical to note that any difference can only be realised if the fundamental principles of recovery are well executed; namely, hydration, fuel restoration and adequate sleep. In addition, an understanding of whether insufficient recovery time is the cause of reductions in function, and hence the individual capacity to train or compete is important. Inevitably, with sufficient time, the body will recover (Fig. 1) without the need for additional interventions beyond the aforementioned fundamentals of recovery. By having a good understanding of the physiological and psychobiological stress that is induced by training and competition, it is possible to discern what interventions might be of use. Despite the known biological differences along the female lifespan, relatively little is known about recovery interventions specifically in female individuals [19]. Nonetheless, the demands of soccer across sexes are relatively similar, so some intimation can be gleaned from the literature, although it must be acknowledged most of the research into the stress-recovery-adaptation continuum is based on male individuals or mixed-sex cohorts.

**Fig. 2:**
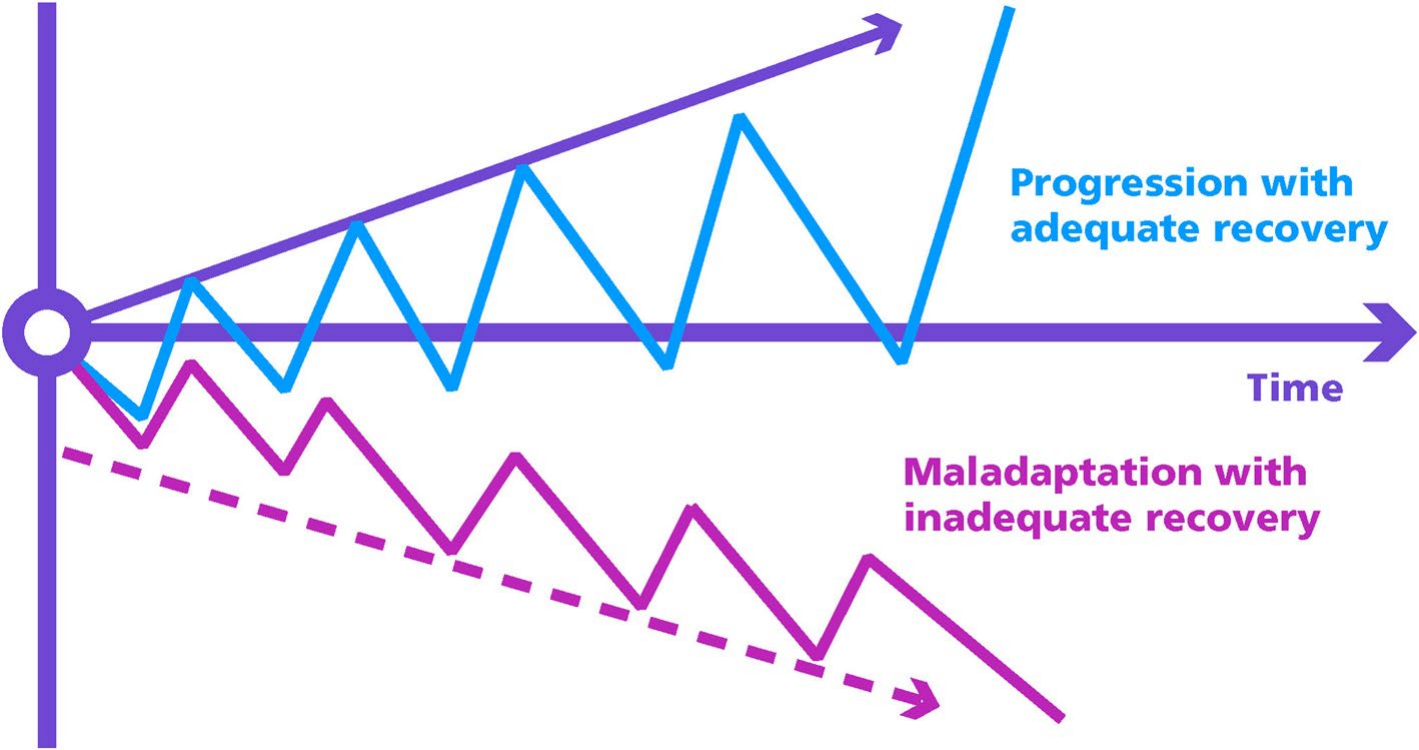
Schematic where recovery and regeneration are adequate to resolve functional decline (*brown line*). In the absence of adequate recovery, there is a cumulative decline in athletic performance (*red line*)

The nature of soccer means that a high metabolic demand [20] and mechanical [21] stressors can lead to muscle damage, muscle soreness, inflammation, and increased production of reactive oxygen and nitrogen species, which ultimately reduce functional capacity [21, 22]. Although this is well demonstrated in male individuals, it has been shown that more pitch playing time and less time between matches (typically < 3 days) in female individuals is also associated with reduced muscle function and physical performance during matches [23–25] and highlights the importance of adequate recovery. This can manifest as reduced muscle function, greater muscle stiffness that leads to a loss of flexibility, reduced skill-based performance and muscle soreness that can last for several days after the stimulus [21, 22, 26]. If athletes train or compete whilst experiencing these symptoms, it is highly likely their performance will be sub-optimal and increase the potential risk for injury [27] because of the reduced ability to express force, and reduced joint position sense and reaction time, for example [28]. It is also known that the risk of match injury for female athletes is approximately six times higher than in training [29], hence increasing the importance of recovery strategies as a tool to reduce the potential for injury. In circumstances such as these, inadequate recovery is potentially the underlying issue and therefore identification of a recovery strategy to accelerate and restore function would be necessary. In a conceptual model of recovery and adaptation (Fig. 2), adequate recovery allows for the maintenance of performance and progression, whereas inadequate recovery has the potential to be maladaptive and ultimately be detrimental to the athlete. In extreme cases, it can lead to non-functional over-reaching or overtraining that can take many months to resolve [30, 31]. This is of particular concern in the female athlete because the unique biological milieu of the menstrual cycle is far from understood in the context of soccer, or indeed most other sports, which can be further magnified by the potential for poor energy management [30]. Under normal circumstances, the restoration of function will occur over time, but the application of a recovery strategy(ies) does have the potential to accelerate the return of function to the basal state sooner and hence place the athlete in a better position to perform to their potential. This is particularly pertinent during times when training is intensified, and competition schedules become congested, and athletes are required to return to their highest level within short time periods.

If the training and competition schedules present challenges, such that complete recovery will not be achieved, strategies could be selected to minimise the deleterious effects on performance. Primarily, the use of recovery interventions should be influenced by: (1) identification of the causes that have the greatest negative effects on performance and the time course of their recovery; (2) the ‘window of recovery’, which is determined by the requirement to next train or compete; and (3) what is required versus what is logistically feasible to achieve; based on the environment, travel constraints, time of day (e.g. evening kick-off), cost benefit, athlete belief and ‘buy-in’ to the intervention(s).

Adequate recovery is extremely important for regeneration, but when is the right time to apply additional recovery strategies to maximise the stress-recovery-adaptation continuum? This dichotomous question has arisen that some recovery strategies might reduce the potential training effect. For example, cold water immersion has been suggested to reduce the cell signalling responses to resistance training [32], whereas it can amplify signalling responses following more endurance-type training [33]. Consequently, many practitioners currently prioritise recovery strategies during tournament situations or periods of fixture congestion or following specific training sessions when adaptation is not the primary focus, but performance in the subsequent round of competition or training session is paramount. This raises the idea of using recovery strategies in a way that is periodised to mirror the demands of the sport and concomitantly balances the need for an adaptive response [34]. However, the literature addressing recovery in female populations is sadly lacking and is largely based on current best practice in other sporting environments or male athletes [35, 36]. What little evidence is available is often underpowered or littered with confounding limitations, which presents a series of exciting opportunities for future research to understand more about the recovery of female soccer athletes.

## Determining the Demands to Inform the Strategy

To prescribe any recovery intervention, it is vitally important to understand the demands of the activity. The demands placed on all soccer players (female and male), from a physical perspective, necessitate multiple intermittent high-intensity activities that consist of repeated changes of direction, accelerations, decelerations, and repeated sprints to transition and press [37]. The accumulated cost of these repeated match-play and training demands is fatigue, which can result in changes in physiological, functional, cognitive and perceptual indices that could affect individual and team performance, and hence overall match outcomes [21, 38].

Fatigue in female athletes is generally greatest immediately following high-intensity passages of play within a game and can accumulate as the match progresses [37]; although fatigue is temporary, recovery can take several days to be resolved [39]. This is further exacerbated during tournament situations where game congestion might be greater, extra time scenarios are required, and hence the magnitude of fatigue is greater and affects physical and skill-based performance [26], which are attributable to both central and peripheral fatigue [22]. Several studies in male athletes reported that the central and peripheral fatigue factors (including perceptual measures) are negatively affected for a minimum of 48 h after a competitive match [21, 40]. In fact, maximal voluntary force capacity is not fully recovered until 72 h after a game in trained male football players [39, 41]. This research was conducted in well-trained male athletes, but the time course of recovery in elite female soccer players is also reported to be ~ 3 days [23, 42], although the magnitude of change in muscle damage indices can be less in female athletes [43]. In female rodents, this has been attributed, in part, to the protective effect of oestrogen, but the picture it is far less conclusive in human female individuals [44]. Notwithstanding, the fatigue profile for female football players is not fully resolved at 48 h post-match [45] and hence poses an issue for training prescription and fixture congestion.

These changes in physiological and fatigue biomarkers have implications on performance, particularly for high-intensity activities, which could also increase the propensity for injury and inhibit long-term athletic development, thus caution is warranted in the event of inadequate recovery. Previous research in male athletes has shown that contributing factors to soccer-related fatigue are derived from the central nervous system (ability to activate the muscle) and peripheral mechanisms (post-synaptic function that reduces the ability for the muscle to operate optimally) because of muscle damage, metabolic stressors and fuel availability [21, 40]. The latter (peripheral factors) persist far longer than central factors and therefore it is intuitive that recovery strategies should preferentially target skeletal muscle. Although the sport remains the same (laws and duration) between biological sexes; elite female athletes show almost complete depletion of muscle glycogen after a match, which is thought to decrease sprint ability in the latter parts of the match and hence anaerobic energy production could be less than male athletes [46]. This could be attributable to lower anaerobic capacity and a sex-related fibre-type difference, where female athletes exhibit a greater proportion of type 1 fibres [47]; but importantly, female athletes with a strong type 1 muscle phenotype tend to have better football-specific endurance performance [48]. In addition, the influence of endogenous and exogenous hormones (e.g. through contraception) can influence the response to exercise [49], particularly at higher exercise intensities [50]; importantly, it seems clear that at least 72 h is required between matches to reduce the potential for performance declines over time [51].

There is a wide array of monitoring tools that could be used to track recovery status in football players. Examples include functional measures of strength and power, speed, agility, and reaction time, wellness, morning heart rate, heart rate variability and sleep (quality and quantity). It is beyond the scope of this review to go into detail of all the potential measures on offer; most football teams will be using a wide and varied array of tools, and variations of those tools, to suit their day-to-day practice. Given the potential importance of these indices, practitioners need to be mindful of the repeatability, and critically the validity of these. For example [52], the agreement between sleep self-reported versus actigraphy measures in high-level female footballers was relatively poor and could vary as much as 2 h when estimating sleep duration for some individuals (self-reporting over-estimating sleep duration). However, the group mean measures are very well correlated (*R*^2^ = 0.88), suggesting both measures are sensitive to change, but the absolute numbers need to be treated with caution. Likewise, in a mixed-sex cohort, heart rate variability was analysed using a smartphone or validated algorithm [53] and showed a very strong relationship (*R*^2^ = 0.85) between measures, but there was unsatisfactory agreement. Collectively, this highlights the importance of measurement standardisation and the limitations of using indices interchangeably. Specific to female athletes is monitoring menstrual cycle length, symptoms, female sex hormones and body temperature. For eumenorrheic female individuals, these all are known to fluctuate across the menstrual cycle [54]. An elongated menstrual cycle length (in a eumenorrheic player not using hormonal contraception) can occur because of excess stress on the hypothalamic-pituitary-ovarian axis (e.g. psychological stress or a relative energy deficiency), and where deviations are significant, medical input might be appropriate. Recovery practices aimed at maintaining energy availability and reducing psychological stress might be helpful in managing these symptoms.

## Is There a Need to Intervene? Getting the Basics Right

**Fig. 3:**
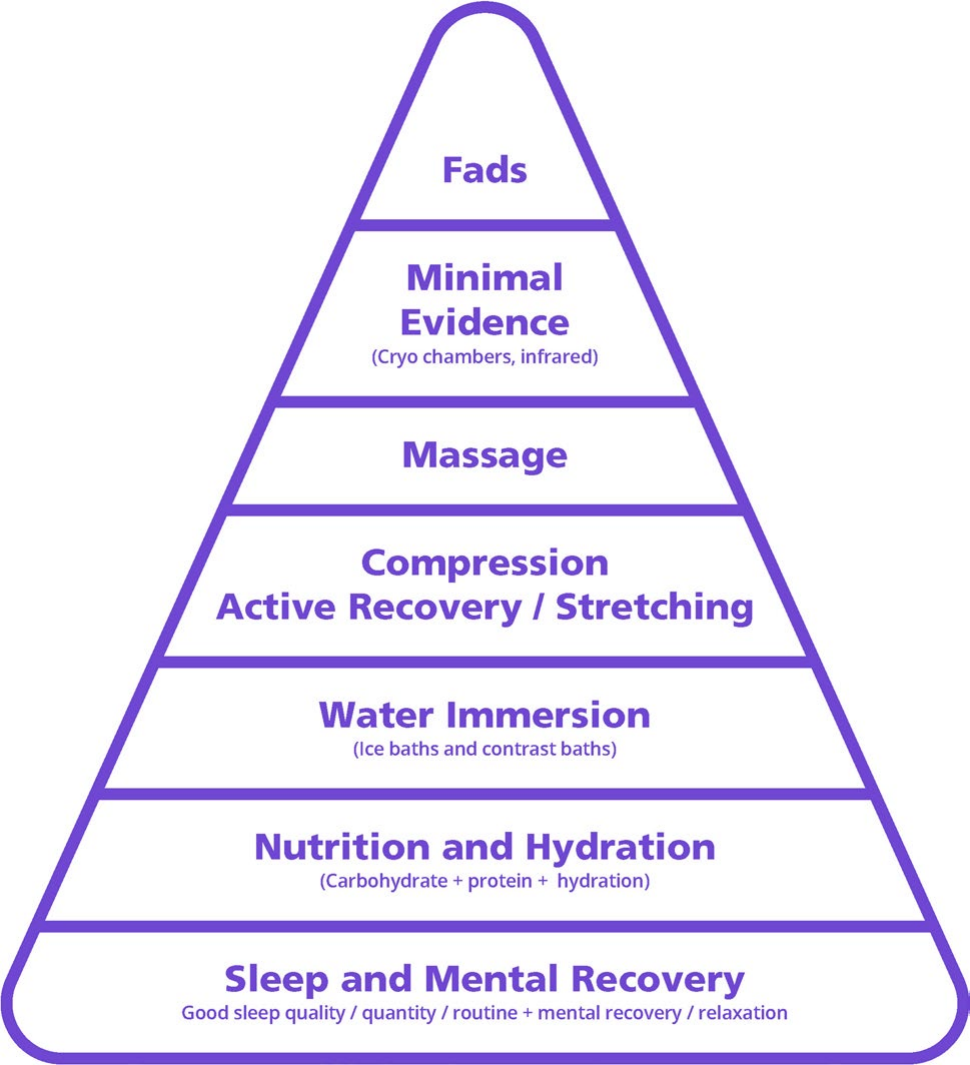
Hypothetical recovery pyramid indicating the priority of recovery strategies, with sleep, mental recovery and nutrition as the suggested foundations. In general, the application of recovery interventions should be made on an evidence base; normally, those with a greater evidence base should be prioritised before those with little or no evidence

Recovery strategies aim to re-establish the psychological, physiological, emotional, social and behavioural manifestations of training and competition. With this in mind, there are two important concepts to consider: (1) has sufficient accumulated training or a competition stimulus occurred to necessitate the implementation of a recovery strategy (in other words, recovery would occur naturally in the desired time frame without intervention), and (2) if so, which recovery strategies should be prioritised? Central to all recovery strategies should be the philosophy of ‘do no harm’, so there is the potential for a negative effect, then, it is unwise to implement that intervention. The recovery pyramid (Fig. 3) outlines the primary recovery strategies that are commonly used in high-performance sports. The recovery pyramid is built on the foundation of sleep, mental recovery and nutrition practices as these areas have the potential for the greatest impact on athletic performance. This foundation can then be built upon by incorporating other strategies such as hydrotherapy, compression and massage, which has received less research attention in female individuals. The top of the pyramid includes strategies that have minimal or no evidence and may be considered ‘fads’ that are momentarily popular.

While it is acknowledged that recovery practices are an important part of the elite athlete’s training programme, the increasing demands placed on female athletes means that time and resources must be used efficiently. Additional recovery strategies should not be programmed when they are not necessary, or at times that could interfere with sleep and rest opportunities. If the training or competition that athletes have participated in has not resulted in sufficient metabolic, structural or mental fatigue, additional recovery might not be necessary, and other activities can be prioritised. Notwithstanding, optimising sleep, hydration and nutrition are considered a ‘non-negotiable’ and should occur as often and as consistently as possible.

This pyramid can be used to provide advice and education to athletes regarding the prioritisation of recovery strategies. For example, the base is the most important aspect to focus on and can have the greatest influence on recovery and performance. The middle section of the pyramid may also be effective and can be appropriate when used in a comprehensive recovery plan. However, these should only be incorporated once the foundation of the pyramid has been addressed. The top of the pyramid includes emerging strategies, which have little or no evidence and, therefore, their effectiveness is unknown or questionable. These strategies should either be avoided or used with the knowledge that they might be ineffective. Importantly, these latter recovery strategies should not be undertaken in place of more effective techniques, thereby decreasing the effectiveness of the overall recovery strategy.

## Importance of Short-Term Recovery Versus Long-Term Adaptation (Periodisation of Recovery)

Traditionally, recovery has always been considered an integral part of adaptation to training, whereby recovery is required following the training stimulus to ensure an athlete does not become excessively fatigued and adapts to the training programme. However, there is currently some debate regarding the role of recovery interventions in potentially decreasing adaptation by blunting the inflammatory and signalling response necessary to promote certain adaptations [55]. These concerns are largely centred around the cell signalling responses in skeletal muscle in response to resistance training. At present, this debate is principally focussed on the use of cold water immersion following resistance training. A conservative approach to this issue might be the removal of additional recovery strategies (which provide a cooling effect) after resistance training sessions because of concerns about negatively influencing strength-related adaptations. However, it must be noted that there is almost no research addressing the molecular responses in elite athletes and none that exclusively focuses on elite female individuals.

**Fig. 4:**
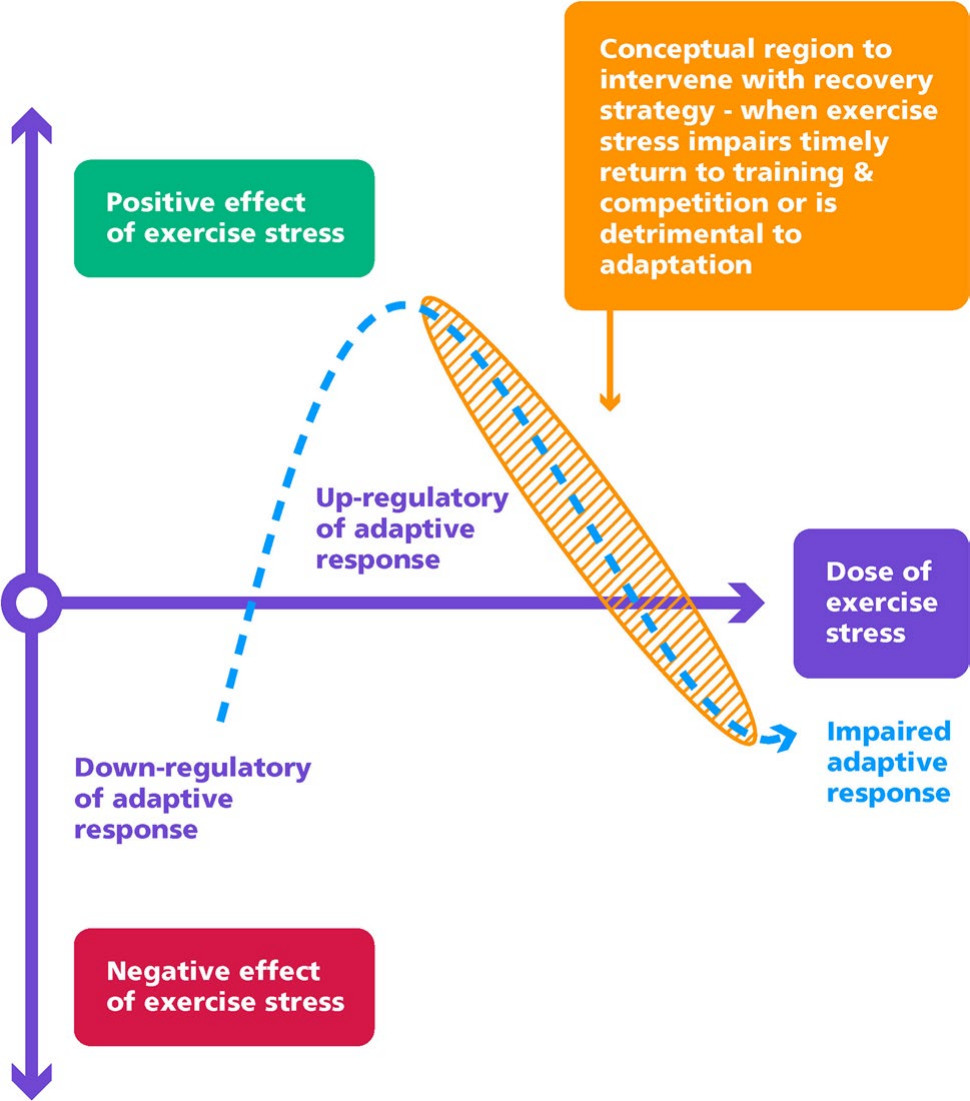
Hypothetical model applying the concept of hormesis to the stress-recovery-adaptation continuum, where exposure to increasing stressors (training or competition) can lead to a positive adaptive response. Further stressors can lead to a maladaptive response where training and non-training stressors becomes too great for the athlete to tolerate

A helpful way to conceptualise this dichotomy is the theory of hormesis (Fig. 4), where exposure to increasing stressors (training or competition) can lead to a positive adaptive response [56]. However, where the further increase in training and non-training stress becomes too great or indeed, too frequent (does not allow for the resolution of fatigue), a maladaptive response might be seen [56, 57] and hence the need to intervene. Although much research is needed, particularly among female athletes, oestrogen is known to have anti-oxidant effects that might serve to shift the hormesis curve, aiding recovery during the follicular phase of the menstrual cycle when oestrogen is high [44].

A more suitable and pragmatic approach would be to periodise the use of recovery [34] for the elite female athlete. In the same way we periodise training and nutrition, consideration should be given to periods in the training programme where recovery interventions can be minimised (e.g. in base endurance training phases) and where they might be emphasised (e.g. during a competition phase or fixture congestion). All things considered, the most important aspect to consider is whether the athlete’s goals are short or long term. Is the current aim to enhance adaptation to training or minimise issues relating to fatigue for better-quality training sessions, competition and/or performance enhancement? For a comprehensive review on periodising recovery in individual and team sports, please refer to a previous review [58].

Considerations for manipulating recovery strategies with the training programme:

- Withholding recovery strategies at certain times, most commonly in the general preparation phase, to maximise adaptation to training (chronic recovery).
- Utilising recovery strategies during the specific preparation phase to prepare for certain training sessions (acute recovery).
- Utilising increased recovery to decrease acute fatigue during the competition phase (acute recovery).
- Incorporating recovery strategies during travel, recovery from injury and managing psychological perturbations (acute and chronic recovery).

## Mental Recovery Considerations

The focus of athlete recovery has traditionally been centred around the restoration of physical attributes such as skeletal muscle function, but there is life above the neck where cognitive or mental aspects can be overlooked. When there is a great deal of focus on physical performance, it is easy to neglect other factors that contribute to human performance. In this section, mental recovery is considered and its importance in the wider recovery of female athletic performance [59, 60]. Previous research has shown that perceived stress and psychological stress induced by the everyday life events of female footballers could impair muscular recovery [61]. Conversely, high levels of physical fatigue have been shown to interfere with both physical and mental recovery [62]. These findings support the potential benefit of an inter-disciplinary approach to recovery, which concurrently targets both physical and mental aspects. Accordingly, while the management of physical recovery remains central, providing information and strategies on mental recovery will undoubtedly be of benefit.

Mental recovery is the process of restoring allostatic balance and the replenishment of cognitive resources [63]. Athletes are routinely exposed to cognitive demands that require sustained mental efficiency through activities undertaken in daily training, competition or other routine tasks such as media appearances that lead to mental fatigue [64]. Given the rise in popularity of women’s football, there is an increasing physical demand on these athletes [7] and concurrent increases in off-pitch demands that include opposition analysis, media involvements, travel to unfamiliar venues, fixture congestion, family and caring responsibilities, and the prominence of contract negotiations. These collective demands have previously been reported by athletes as inducers of mental fatigue [64]. One contemporary example is gendered harm that female athletes might experience through social media; the subsequent emotional regulation required in response to negative exposures can impose greater cognitive demands [65]. Moreover, social-cultural factors position many female athletes to experience greater cognitive demands because of factors relating, but not limited to, lower wages resulting in the need for additional work or concurrent academic studying, as well as taking on traditional caring roles [66]. Accordingly, female athletes could experience greater cognitive demands additional to those resulting from their training and competition demands as a footballer, and therefore, the implementation of evidence-based approaches to enhance mental recovery is essential to female athlete recovery.

Although there is a clear requirement for athlete mental recovery, the evidence regarding the efficacy of specific interventions for athletic populations is scarce and inconsistencies in research approaches exist. Accordingly, this limits the capability to provide guidelines and specific protocols to enhance athlete mental recovery. However, prior investigations have explored the management of mental fatigue and mental recovery in athletic populations from the perspective of three overarching approaches. These approaches include (1) acute enhancement of mental recovery and restoration [59], (2) acute mitigation of mental fatigue to minimise potential performance impairment [67] and (3) deliberate periodised inducement of mental fatigue, interspersed with an opportunity for mental recovery [68]. Focusing on the enhancement of mental recovery, practical strategies that have been explored to enhance mental recovery include breathing techniques, exposure to restorative environments, specific music frequencies (i.e. binaural beats) and power naps [69]. For breathing techniques, systematic breathing techniques, such as resonance frequency and biofeedback breathing protocols, for a duration of 20–25 min could be beneficial to cognitive restoration [69]. Exposure to restorative environments, such as scenes of national parks, mountainous areas, oceans or lakes, can enhance mental recovery [70]. This exposure may be implemented through various techniques, including physical immersion (i.e. visiting the restorative location) mental imagery, photographic viewing [71] or exposure via virtual reality [72]. Further research is required to make clear recommendations on the protocol duration for each of these techniques for female individuals; however, exposure bouts of approximately 15 min have demonstrated efficacy, supporting potential for use in athletic settings. Listening to specific frequencies of music could also be mentally restorative. A 6-min track, with 165 Hz in the left and 179 Hz in the right ear, resulting in a binaural beat in the beta range of 14 Hz, has been shown to enhance cognitive control [73]. Accordingly, timely exposure to this music frequency, where mental restoration and control is desired could also be of benefit. Aligned with the sleep literature, athletes can also benefit from a 20–90 min nap between the hours of 13:00 and 16:00 with regard to improving cognitive restoration and psychological state [74]. Furthermore, while not a direct recovery method per se, strategies that acutely mitigate the potential experience of, or subsequent negative impact of, mental fatigue during performance could be advantageous. These acute techniques can be implemented immediately prior to, or during performance, and include caffeine intake, exposure to specific odours such as menthol and citral, and performance feedback [67]. Beyond these acute strategies to promote mental recovery, adequate sleep quantity and quality are essential to enable cognitive restoration and improve subsequent performance [75]. Therefore, strategies to promote good levels of sleep hygiene are critical for athlete recovery.

It is acknowledged that challenges to the implementation of mental recovery include a lack of practitioner knowledge and confidence in the implementation of a strategy, time constraints and limited resources [60]. Therefore, to promote athlete mental recovery, it is imperative to provide easily accessible and time-efficient strategies. Increasingly, individualised mental recovery techniques, such as guided breathing interventions, are accessible through publicly available or custom-made low-cost applications as well as being integrated into wearable technology applications. Recent technological advances make the application and affordability of interventions, including virtual reality, more accessible and have progressed the feasibility of implementing techniques for mental restoration.

Despite the inclusion of female participants in select mental recovery studies, [69, 76] a majority of research investigating psychobiological recovery has been conducted with male individuals. The limited involvement of female participants in these types of studies further underlines concerns regarding the under-representation of female athletes in broader sports science and sports medicine research [35, 36]. Importantly, prior laboratory-based evidence demonstrated differences in cognition response between female and male athletes, highlighting the need for female-specific data [77]. Furthermore, the cyclic fluctuations in ovarian hormones and complex changes, which occur across a female individual’s lifespan, could also influence aspects of cognition and mental recovery in female athletes [78, 79]. Collectively, there is a need for investigations designed to evaluate the suitability of mental recovery approaches in female athletes that account for their unique physiology and psychobiological responses that can be used to inform practice. However, practitioners need to be cognisant of the current need for mental recovery in female athletes and actively employ strategies to supplement and complement physical recovery processes.

## Education and Athlete Belief in Delivering Recovery Strategy Efficacy

Despite an increase in the knowledge and awareness of sleep and recovery, many athletes engage in behaviours that can (1) hinder night-time sleep (social media, computer games, streaming platforms for movies/TV shows/games, caffeine intake) and (2) do not engage in optimal recovery practices. Traditionally, an education focus has been placed on training, but information for athletes in the area of recovery, physiology and psychology, is just as important. Education will help engage athletes and coaches to ‘buy-in’ to strategies resulting in improved recovery practices. Providing simple practical guidance on the benefits of recovery and how it will assist their performance is highly advisable. This can also provide the opportunity to inform athletes regarding current best-practice techniques as well as those that should be avoided. In mature experienced athletes, it can also be beneficial to provide education regarding mechanisms of recovery to help them choose strategies based on scientific information rather than fads, anecdote or sales pitches.

The belief in the effectiveness of a recovery intervention can be powerful and have positive biological and performance outcomes. Educating the athletes regarding the scientific evidence-based effects of a recovery strategy can increase the belief effect. Therefore, providing scientific evidence combined with powerful belief effects provides the basis for both optimising performance and maintaining ethical standards [80]. Furthermore, a growing body of evidence indicated that recovery is related to individual preferences and perceptions of the intervention [81]. Accordingly, it is important to recognise and, where necessary, manage the influence of belief and the power of placebo effects for a successful recovery strategy to be implemented. This raises the need to achieve coach and athlete buy-in to any intervention, and the challenge to balance an evidence-based approach with the beliefs and expectations of coaches and athletes [80]. In cases where an athlete believes in a particular recovery strategy despite a lack of supporting scientific evidence, the demand on resources (financial, time, effort), the cost (i.e. what is sacrificed by engaging in a particular strategy), and most importantly, the potential for harm or a negative performance effect, must be evaluated. On balance, if there is no negative effect and the athletes believe in the intervention, then, even considering the little supporting evidence, the intervention might be beneficial. It is therefore important to use recovery strategies on a very individualised basis to ensure the most benefit can be gained from an intervention.

Behavioural science can help play a role in enhancing our ability to make changes to athlete sleep behaviours, but also other aspects of athlete recovery, health and well-being. It may be beneficial to identify the target behaviour, understand the psychological determinants of the behaviour and develop behavioural change interventions based on scientific evidence [82]. For example, understanding what drives the athlete in terms of long-term goals as well as why they are engaging in specific activities that could be negatively influencing sleep. It is important to understand what the individual’s drivers of behaviours are, so these can be targeted. One way to support this is through performance lifestyle support that can help mentor and coach female athletes’ health and well-being by facilitating personal and professional development [83], which is particularly important in younger female individuals. In addition, understanding what environmental or scheduling interferences are imposed on the athlete could help identify a target area for a positive change [75, 84]. For example, early morning and late evening training sessions, sharing rooms and team meetings before bedtime could interfere with sleep, but this could easily be modified by the staff working with the team. Further, if psychological techniques such as stress management, meditation or progressive muscle relaxation are suggested as a means to assist the athlete, then the athlete must be provided with the skills, knowledge and access to maximise these techniques.

## Specific Considerations Around Menstrual Cycle Symptoms

A host of menstrual cycle symptoms are known to occur in female athletes (see other sections), and these can disrupt training and performance, with implications for recovery priorities and monitoring data. The most common symptoms reported are mood changes including motivation and anxiety, an increase in appetite/cravings, breast pain/tenderness, lower back and general muscle soreness, and tiredness/fatigue [15, 85]. Few athletes experience no symptoms at all; however, there is a wide variation in the severity and therefore the impact of such symptoms, meaning that a few players in a team could be significantly affected by symptoms and a few might not experience symptoms at all. The more symptoms experienced, the greater the probability of missing training, using non-steroidal anti-inflammatory drugs and missing competition [15]. So far, very few studies have specifically reported on the prevalence of menstrual cycle symptoms in soccer, but in the general population and exercising populations up to 90% of female individuals do experience some symptoms [15].

Specific menstrual symptoms have been associated with inflammation, at least in non-athletes [14, 86], and it is possible that anti-inflammatory strategies (primarily sleep and diet), together with reducing proinflammatory behaviours, could be particularly useful to reduce these symptoms. Gold et al. [86] linked changes in mood, stomach cramps, back pain, cravings, bloating/weight gain and breast pain to increased C-reactive protein, a generic inflammatory blood marker. One research group examined pre-menstrual syndrome and its association with inflammation and anxiety. Defined as symptoms typically occurring in the luteal phase between ovulation and menstruation (where progesterone is the dominant sex hormone), pre-menstrual syndrome is associated with increased inflammation, and fluctuations in neurotransmitters [87, 88] in the central nervous system, leading to transient neuropsychological changes, which might result in anxiety and panic. Accordingly, it is important to be mindful that female athletes are reported to be twice as likely to experience anxiety and associated psychological disruption as their male counterparts [89]. Given that scenarios occurring in elite sport, particularly at the international competition level, are also associated with high-pressure and subsequent psychological stress, it follows that recovery strategies emphasising psychological well-being and recovery, such as biofeedback breathing, napping or exposure to restorative environments, can be particularly important for female athletes. Recovery strategies can help reduce menstrual cycle symptoms, either objectively or subjectively. The importance of these strategies can be heightened where symptoms correspond with additional physical and psychological stressors such as fixture congestion, international travel and championships, and contract negotiation, collectively leading to a ‘perfect storm’ of stressors. Consequently, practitioners should be mindful of players’ menstrual cycle phase and individual susceptibility to symptoms. This will enable bespoke interventions to be appropriately scheduled according to training and playing programmes, but with due consideration to menstrual cycle symptoms. Although there is little-to-no evidence base for an approach of this nature, it makes teleological sense to consider scenarios where symptoms coincide with strenuous training, or fixtures (congestion) and consequently be catered for with interventions that might mitigate the milieu of training and non-training stressors.

## Potential Application of Recovery Strategies

**Fig. 5:**
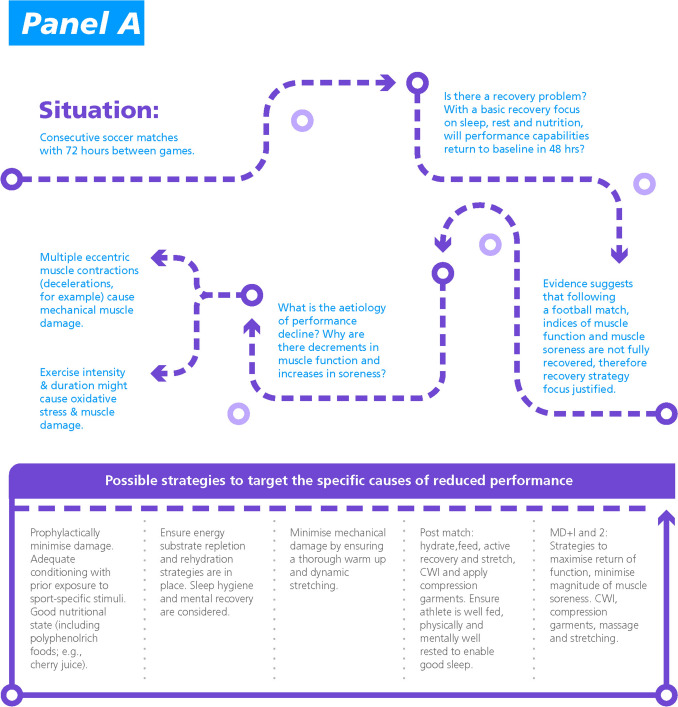

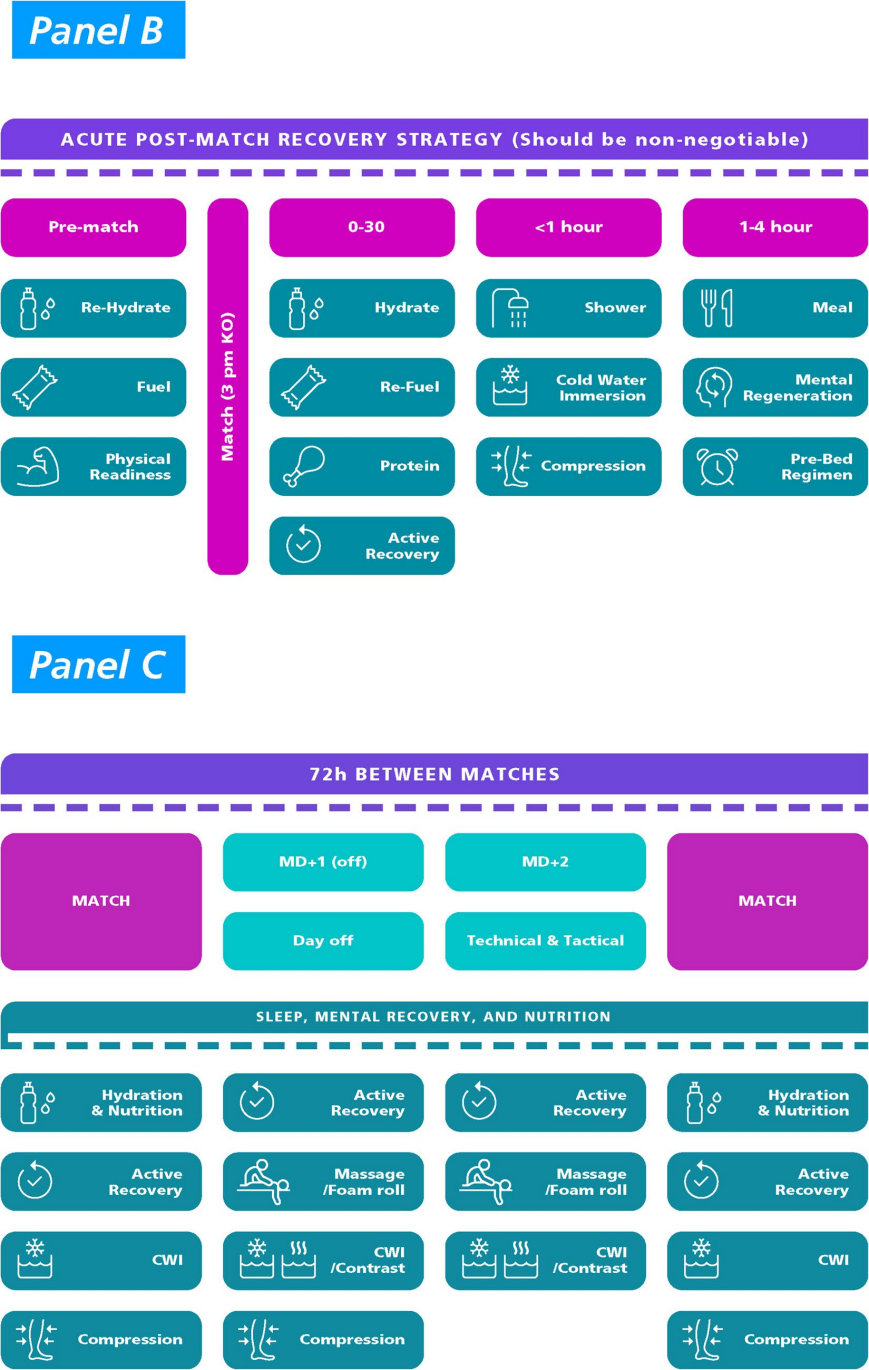
Representation of strategies that could be used to accelerate recovery. The *upper panel* provides context to the case study and the route of decisions that should be considered in applying a strategy. The *middle panel* provides a series of acute interventions that should be prioritised immediately following a match. The *lower panel* provides a series of interventions that might be considered in preparation for a match 72 h (hrs) later. *CWI *Cold water immersion

The schematic (Fig. 5) represents a scenario where there is a requirement to prepare, play, recover, and be prepared for a second match within 72 h of the first (Panel A). Panel B illustrates the acute post-match strategies that could be applied immediately after the match. Panel C provides an illustration of the strategies that could be applied in the period between games to promote recovery. Importantly, this schematic presents one way in which recovery strategies could be applied based on the demands of the match and the consequences that are likely to be precipitated. Of note, the strategies are based on an evidence base and can be implemented relatively simply.

## Summary

**Table:** 

• Understand your athlete and the training and competition stressors causing reductions in performance and delaying recovery. All female athletes will react differently to the stressor and have different menstrual cycle symptoms. Knowledge of the athlete will then help inform what strategy might be used.
• Determine if a strategy is necessary or whether recovery can be attained without intervention. Contemplate the relative importance of short-term recovery and long-term adaptation; consider how the idea of hormesis might be applied. Do not blindly apply strategies if it is not necessary.
• Consider the importance of both mental and physical regeneration. Athlete belief and ultimately, buy-in should not be underestimated.
• Whilst there is some useful research for female athletes that can be applied, future research should combine multidisciplinary (physical and mental) approaches. In addition, studies that examine isolated mechanistic and/or performance-focussed approaches to elucidate the impact of recovery strategies on recovery and adaptation in female athletes are in woefully short supply and are desperately needed.

To conclude, optimising recovery will likely positively influence training, adaptation and performance in female football players. The increasing physical, mental and societal demands placed on female athletes combined with their unique hormonal profiles, indicate that careful consideration and periodisation of recovery is increasingly important. However, because of insufficient research, female-specific interventions are lacking and are typically based on data from male athletes; some of these data might be useful but are often fraught with limitations when applying to female athletes. Notwithstanding, female athletes should prioritise foundational recovery strategies such as sleep, mental recovery and nutrition to optimise recovery and ultimately improve performance. Understanding individual athlete menstrual symptoms and phases of the cycle and other biological idiosyncrasies, mental state, external stressors and personal challenges will place practitioners in a better position to apply additional strategies to address the milieu of potential accumulated playing and non-playing stressors.
